# Molecular mechanisms of Y chromosome loss and *UTY* gene activity

**DOI:** 10.2144/fsoa-2024-0031

**Published:** 2024-05-15

**Authors:** Berjas Abumsimir, Talal Al-Qaisi, Sima Zein

**Affiliations:** 1 Molecular Medicine Team, Faculty of Allied Medical Sciences, Al-Ahliyya Amman University, Jordan

**Keywords:** acute myeloid leukemia, loss of Y chromosome, RUNX1, T cell acute lymphoblast leukemia, UTY gene

The Y chromosome is considered a sex-determining chromosome, this is the prevailing stereotype. Large parts of the Y chromosome are also considered genetically as noncoding regions and contain few active genes compared with other chromosomes. However, the roles of some genes in this chromosome require further exploration, especially regarding the development of serious diseases during male life.

The loss of the Y chromosome (LoY) has been linked to many serious health problems, including cancer. However, the precise effect of the LoY at a molecular level is still a matter of speculation. This commentary aims to initiate a discourse on the molecular mechanisms underlying the effects of LoY while also examining the biological and clinical observations related to these mechanisms.

The *UTY* gene (also known as *KDM6C* or Yq11.221) is a paralog of *UTX* (*KDM6A*) on chromosome X. *UTY* encodes a 149.5 kDa protein that is known for its low demethylation activity [1]. At present, we do not have a full portrait of UTY's role in the cell, so we have used the best available evidence to reveal the impact of *UTY* gene absence.

The UTY protein has unique tetratricopeptide repeats (TPRs), that serve as protein–protein interaction motifs. UTY binds effectively with TLE1, a protein known for its transcription factor repression activity. The binding occurs exactly at the first three TPRs of UTY, with evidence suggesting that the N terminal half of TLE1 is sufficient to mediate UTY binding [2]. UTX or UTY/TLE1 binding follows in the same manner. All observations indicate that this mechanism is fundamental and seems to be conserved not only across Mammalia but also in invertebrates. Previous conclusions thought UTY/TLE1 binding may strengthen the interaction between TLE1 and DNA-binding proteins [3].

Subsequently, the UTY/TLE1 complex is recruited by transcription factor RUNX1 (*AML1* is one of the aliases). The binding occurs on runt domain (RD). RD terminates in a common pentapeptide, VWRPY, which serves to recruit the corepressor. UTY does not bind directly to RUNX1 but instead binds to TLE1, which binds physically to RUNX1 (forming the CBF complex), which further binds with β-catenin [4].

RUNX1 is recognized for its important role in hematopoiesis through the Wnt/β-Catenin signaling pathway. The CBF complex (composed of RUNX1 and RUNX2) contributes to this pathway by modulating the activation/inactivation of target genes [^5-7^]. At this point, there is a margin of conjecture that the T-cell receptor (TCR) T-cell factor (a transcription factor that plays an important role during the T-cell development and differentiation), is engaged somewhere in the pathway heading to differentiation and maturation of hematopoietic cells. It is important to note that TLE1 participates in other signaling pathways in addition to Wnt/β-Catenin, including NOTCH and Hedgehog signaling pathways.

There is also another route of UTY/TLE1 complex coregulation, this time binding LEF1, a transcription factor that mediates transactivation of TCR enhancers, continuing the way of TCR corepression [4,8,9].

If we start reviewing the evidence that supports this vision, recent findings conclude that cancer cells with LoY, alter T cell function and promote T-cell exhaustion [10]. As mentioned above, the UTY/TLE1-RUNX1 complex plays a role in regulating T-cell receptors, and RUNX1 inactivation (in the case of UTY absence) causes a production of immature T cells, which could later manifest as exhaustion.

In addition to the previously described RUNX1 activity in stem cells, embryogenesis and other physiological roles, it is conceivable that RUNX1 could be active in a discontinuous manner in response to male life span developments. This explains many health problems which we will discuss later.

RUNX1 alterations, whether due to loss, mutation or translocation, is associated with the immaturation of the lymphoid/myeloid cells [7]. Similarly, the absence of the UTY/TLE1 complex will produce the same effect due to the loss of repression mechanisms. RUNX1 loss will produce a RUNX1-ETS fusion protein, identified as acute myeloid leukemia (AML) [11]. Together, *UTY* absence plus the loss or mutation of its paralog *UTX* gene on the X chromosome will alter RUNX1 activity.

While UTY protein was initially thought to compensate for the absence of *UTX* (*KDM6A*), recent evidence indicates that this compensation is limited [12]. Based on our discussion above, together with loss/mutation of UTX and loss of UTY (due to entire Y chromosome loss); are two simultaneous conditions for the inactivity of certain roles in RUNX1.

The binding of UTY/TLE1 with RUNX1 is believed to be necessary for strengthening DNA binding, and TLE1/UTY binding with RUNX1 must have a repressor effect [4]. We agree with this conclusion in some cases particularly concerning the time-restricted activity of RUNX1 during the male lifespan, it is necessary to wait for the right time to act (e.g., embryonic development, puberty). This also applies to the semi-continuous process of differentiation of hematopoietic cells. Loss of the Y chromosome could occur at any time in the male lifespan, highlighting the immediate impact on cellular processes.

The involvement of the UTY/TLE1 complex in RUNX1 regulation is supported by the evident role of TLE1 in hematopoiesis, epithelial differentiation and neuron development [13] as well as the observation of notable expression of UTY in stem cells in comparison with other tissues. The latter observation was discovered when researchers tried to identify the UTY epitope responsible for the stem cell graft rejection from males to females [14].

T-cell acute lymphoblast leukemia (T-ALL) is certainly linked to RUNX1 activity. The incidence sex ratio of T-ALL is skewed toward males (one female: three males), while acute myeloid leukemia AML shows a slight male predominance with an incidence sex ratio of 1.3 male:1 female [15]. This difference may be attributed to the number of *UTX* (*KDM6A*) gene copies (two in females: one in every X chromosome, and one in males on the X chromosome). The increased incidence in males may be due to insufficient compensation in the absence of a second *UTX* (*KDM6A*) copy, while in females *KDM6A* activity can be easily compensated through inactivation of the other copy of the same gene.

In inheritance fundamentals, the entire X chromosome in females should be silenced, but there is strong evidence indicating that 15% of inactivated X chromosomes escape inactivation, and *KDM6A* is one of them [16]. Here the T-ALL and AML case ratio difference between males and females are justified. The other famous example is bladder cancer, with a male: female ratio of 1:4 [17], which could be annexed to the previous justification.

The association between AML and T-ALL on one hand and LoY on the other hand in children can be verified by the karyotyping tests. This will be informative evidence of the RUNX1-UTY/TLE1 coregulation mechanism. It is important to note that while we attribute some incidence differences to LoY and the proposed absence of UTY/TLE1, we acknowledge that other factors contribute to the development of these tumors.

The possible association between UTY and RUNX1 is supported by UTY H3K27me3 activity, a state unique to stem cells [18]. Additionally, evidence from cellular components suggests the presence of UTY/TLE1 and RUNX1 in the nucleus in normal conditions. Another important observation is that TLE 1 inactivation contributes to the development of hematological malignancies [14].

Concerning the hormonal effect of tumor initiation (which has its defenders and supporters among clinicians and researchers), AML, T-ALL and bladder cancer have never been linked previously to hormonal effects or therapy, in comparison to classically sex-linked tumors. Moreover, the T-ALL, AML and spectrum of bone tumors incidences records usually among children who do have not any significant hormonal effects yet.

The accumulated evidence of linkage between neurodegenerative diseases and LoY, combined with the role of RUNX1 in pain neuron development [19], and according to our description of UTY/TLE1-RUNX1 coregulation; such linkage is still not clear to us. For example, let us consider the equal incidence ratio of Alzheimer's in males and females. Two explanations are possible here: either UTY/TLE1 does not participate in such a mechanism, or the linkage between Alzheimer's and LoY should be reinvestigated.

RUNX1 has another role in epithelial tissue to control hair follicle development from telogen to anagen through activating the Wnt signaling pathway and LEF1 levels [20]. Integrating the UTY/TLE1 complex into this role of RUNX1 could potentially explain the differing ratios of hair loss between males and females, despite the multifactorial nature of hair loss causes.

This commentary explored the indirect linkage between UTY and RUNX1 by collecting previous evidence and analysis of the physiological role of the two gene outcomes. However, our recent knowledge is still insufficient. To better understand the effect of LoY on male health, further investigation into their physiological roles and interactions is necessary. Moreover, exploring UTY protein interactions and demethylase activity, as well as investigating periods of RUNX1 activity cessation could provide valuable insights into the implications of LoY on male health. What's more, the third family member *KDM6B* (with *KDM6C* and *KDM6A*) may have a suspected compensation mechanism that warrants further investigation. Given its location on autosomal chromosome 17, the *KDM6B* gene may be a field of interest in evolutionary studies concerning the Y chromosome.
